# The Effect of Anesthetic Technique on Survival in Human Cancers: A Meta-Analysis of Retrospective and Prospective Studies

**DOI:** 10.1371/journal.pone.0056540

**Published:** 2013-02-20

**Authors:** Wan-Kun Chen, Chang-Hong Miao

**Affiliations:** Department of Anesthesiology, Shanghai Cancer Center and Cancer Institute, Shanghai Medical College, Fudan University, Shanghai, People’s Republic of China; Università Vita-Salute San Raffaele, Italy

## Abstract

Animal models have shown that regional anesthesia (combined with or without general anesthesia) would attenuate the surgical stress response by preserving immune function and result in better long-term outcome. In order to test the hypothesis that cancer patients who had surgery with epidural anesthesia (EA) would have better outcome (either overall survival [OS] or recurrence-free survival [RFS]) than those who were general anesthesia (GA), we performed this meta-analysis. By searching relevant literature, a total of 14 studies containing 18 sub-studies (seven in OS analysis and eleven in RFS analysis) were identified and meta-analyzed. Adjusted hazard ratios (HRs) with 95% confidence intervals (CIs) were used to assess the strength of association. For OS, the random-effects model was used to analyze the data and demonstrated an OS benefit in favor of EA compared with GA alone (HR = 0.84, 95% CI 0.74–0.96, P = 0.013). The influence analysis showed the robustness of the results. Specifically, a significantly positive association between EA and improved OS was observed in colorectal cancer (HR = 0.65, 95% CI 0.43–0.99, P = 0.045). For RFS, the random-effects model was used to analyze the data and no significant relationship between RFS benefit and EA (HR = 0.88, 95% CI 0.64–1.22, P = 0.457) was detected. In conclusion, our meta-analysis suggests that epidural anesthesia and/or analgesia might be associated with improved overall survival in patients with operable cancer undergoing surgery (especially in colorectal cancer), but it does not support an association between epidural anesthesia and cancer control. Prospective studies are needed to determine whether the association between epidural use and survival is causative.

## Introduction

Most surgery needs a procedure of anesthesia. Addition of epidural anesthesia and analgesia to the general anesthesia (referred to as EA in this paper) may be beneficial compared with general anesthesia (GA) because epidural anesthesia may provide better postoperative pain relief, reduce incidence of side-effects, and decrease the possibility of occurrence of immunosuppressive factors such as anesthetic drugs and opioids.[1]–[3] Regional anesthesia largely prevents the neuroendocrine stress response to surgery by blocking afferent neural transmission from reaching the central nervous system and activating the stress response and by blocking descending efferent activation of the sympathetic nervous system, so few opioids (if any) are needed. [3] Animal models have shown that regional anesthesia (combined with or without general anesthesia) would attenuate the surgical stress response by preserving immune function and result in better long-term outcome. [4].

To a large extend, the opposing forces of immune surveillance and a tumor’s ability to spread determine whether local recurrence or metastasis occurs, even, good or poor survival. By reducing the surgical stress response and significantly reducing exposure to opioids (though morphine shows an antiangiogenic potential [5]), regional anesthetic techniques (under most circumstance, combined with general anesthesia) may suppress immune function less than opioid analgesia. Consistent with this hypothesis, some, though not all, studies have shown that there is an association between improved outcomes after cancer surgery and regional anesthetic techniques.[2], [3], [6]–[8] To further improve our hypothesis that epidural anesthesia and/or analgesia (combined with or without GA) might be associated with reduced cancer recurrence after oncological surgery and subsequently improve overall survival, we performed this meta-analysis.

## Methods

### Study Identification and Data Extraction

Relevant studies were searched in the PubMed, Medline, and Web of Science database (updated to August-1, 2012) using the following search terms: (“regional anesthesia” or “epidural anesthesia” or “general anesthesia” or “anesthetic technique”) and (“recurrence” or “metastasis” or “survival”) and (“cancer” or “carcinoma”). Only those published in English language were included; we did not define the minimum number of patients to be included for meta-analysis. Either abstract or full text paper was eligible. By this search strategy, 731 papers were identified. After review their titles, 135 were crudely identified to be possibly eligible. All the 135 abstracts were read; if potentially eligible, the full text paper was retrieved and read. The inclusion criteria were: (i) comparing the effect of EA (combined with or without GA) on survival or recurrence with that of GA in cancer surgery, (ii) independent retrospective or prospective study, and (iii) with sufficient available data to estimate a hazard ratio (HR) with 95% confidence intervals (CIs). Whenever available, we extracted the HR adjusted for other prognostic or confounding factors as most other studies suggested. [9], [10] The adjusted HRs rather than crude odd ratios or relative risks might be more reliable to reflect the effect of anesthetic technique on survival in human cancers. After reviewing full text of all the potentially eligible papers, we identified 14 eligible studies for this meta-analysis. The following variables were extracted from each study if available: first author’s surname, publication year, cancer type, design type, numbers in EA group, number in GA group, and HR with 95% confidence intervals (CIs) of treatment outcomes. The information was collected independently by the two authors (C.W.K. and M.C.H.), and any discrepancy were resolved by discussion. The study quality was assessed using the 9-star Newcastle-Ottawa Scale (The Newcastle-Ottawa Scale for assessing the quality of nonrandomized studies in meta-analyses. Ottawa, Canada: Dept of Epidemiology and Community Medicine, University of Ottawa. http://www.ohri.ca/programs/clinical_epidemiology/oxford.htm. Accessed 2013 Jan 1). We also followed the PRISMA statement for reporting systematic reviews that evaluate health care interventions. [11].

### Statistical Methods

This systematic review and meta-analysis was planned, conducted, and reported in adherence to the standards of quality for reporting meta-analysis. [12] For each study, HR with its 95% CIs was retrieved from paper to estimate the association between EA and survival outcomes. The heterogeneity among studies was assessed by Cochran chi-square Q statistics and I-square statistics, which determined the appropriate use of either fixed-effects (Mantel-Haenszel method) or random-effects (DerSimonian and Laird method) model. Heterogeneity was considered as either a P-value <0.05 or I-square >25%. [13] The potential publication bias was examined visually in a funnel plot of ln[OR] against its standard error (SE), and the degree of asymmetry was tested using Egger’s test. We also showed the meta-analytic results stratifying by cancer type and design type. Influence analysis (sensitivity analysis) was conducted by omitting each study to find potential outliers. All of the statistical analyses were performed using Stata/SE version 10.0 (Stata Corporation, College Station, TX).

## Results

### Basic Characteristics

**Figure 1:**
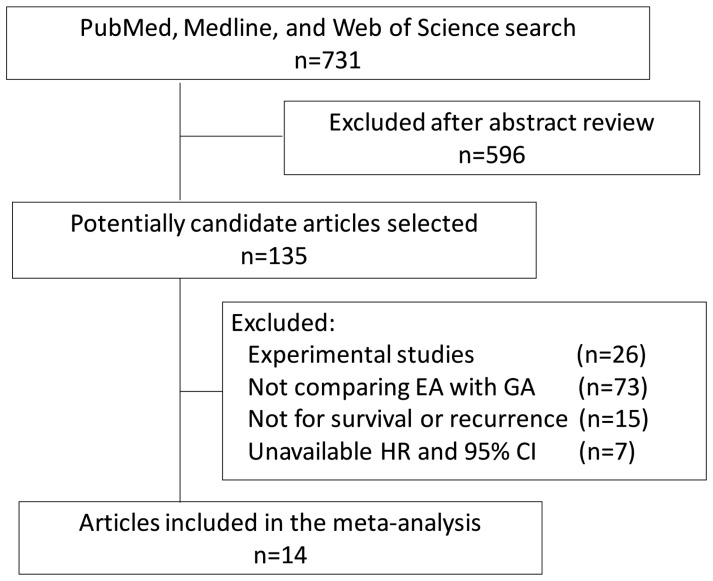
PRISMA flowchart. EA, epidural anesthesia; GA, general anesthesia.

The literature search flowchart is shown in Figure 1. We identified 14 eligible studies for this meta-analysis[2], [3], [6]–[8], [14]–[22] (Table 1), consisting of about 35,000 cases in the GA group and about 12,000 cases in the EA group. There were two study end points: one was overall survival (OS) and the other was recurrence-free survival (RFS). RFS was calculated from surgery to the first occurrence of disease progression or relapse due to the primary cancer; OS was calculated from surgery to the death from any cause.

**Table 1:** Characteristics of eligible studies for meta-analysis.

Author	Year	Cancertype	Designtype	Survival	Studyquality*	GA No.	EA No.	HazardRatio	95% CI
Christopherson-I [6]	2008	Non-metastatic colon cancer	Prospective	Overall survival	7	92	85	0.216	0.065–0.718
Christopherson-II [6]	2008	Metastatic colon cancer	Prospective	Overall survival	7	92	85	0.699	0.395–1.236
Gupta-I [14]	2011	Colon cancer	Retrospective	Overall survival	6	93	360	0.82	0.30–2.19
Gupta-II [14]	2011	Rectal cancer	Retrospective	Overall survival	6	93	295	0.45	0.22–0.90
Lin [15]	2011	Ovarian cancer	Retrospective	Overall survival	6	37	106	0.824	0.699–0.930
Myles [16]	2011	Abdominal malignancies	Prospective	Overall survival	7	216	230	0.95	0.77–1.18
Cummings [17]	2012	Colon cancer	Retrospective	Overall survival	8	32,481	9,670	0.91	0.87–0.94
Exadaktylos [3]	2006	Breast cancer	Retrospective	Recurrence-free survival	6	79	50	0.21	0.06–0.71
Biki [2]	2008	Prostate cancer	Retrospective	Recurrence-free survival	6	123	102	0.43	0.22–0.83
Gottschalk [8]	2010	Colorectal cancer	Retrospective	Recurrence-free survival	7	253	256	0.82	0.49–1.35
Ismail [18]	2010	Cervical cancer	Retrospective	Recurrence-free survival	6	69	63	0.95	0.54–1.67
Luo [19]	2010	Colon cancer	Retrospective	Recurrence-free survival	7	931	182	1.326	0.940–1.871
Tsui [7]	2010	Prostate cancer	Prospective	Disease-free survival	6	50	49	1.33	0.64–2.77
Wuethrich [20]	2010	Prostate cancer	Retrospective	Progression-free survival	7	158	103	0.45	0.27–0.75
Myles [16]	2011	Abdominal malignancies	Prospective	Recurrence-free survival	7	216	230	0.95	0.76–1.17
Oliveira Jr [21]	2011	Ovarian Cancer	Retrospective	Recurrence-free survival	7	127	55	0.37	0.19–0.73
Lai [22]	2012	Hepatocellular carcinoma	Retrospective	Recurrence-free survival	7	117	62	3.66	2.59–5.15
Cummings [17]	2012	Colon cancer	Retrospective	Recurrence-free survival	8	31,099	9,278	1.05	0.95–1.15

* evaluated by the 9-star Newcastle-Ottawa Scale.

Regarding the OS, there were seven sub-studies involved,[6], [14]–[17] and four of them showed significant relationship between improved OS and EA. [6], [14], [15], [17] Five sub-studies were with regard to colon or rectal cancer.

Regarding the RFS, 11 sub-studies including more than five types of cancers (breast, prostate, colorectal, cervical, ovarian cancer, hepatocellular carcinoma, etc.) were included for meta-analysis.[2], [3], [7], [8], [16]–[22] The original results were mixed, with four of them reported positive associations between EA and improved RFS.

### Association between EA and OS

**Figure 2:**
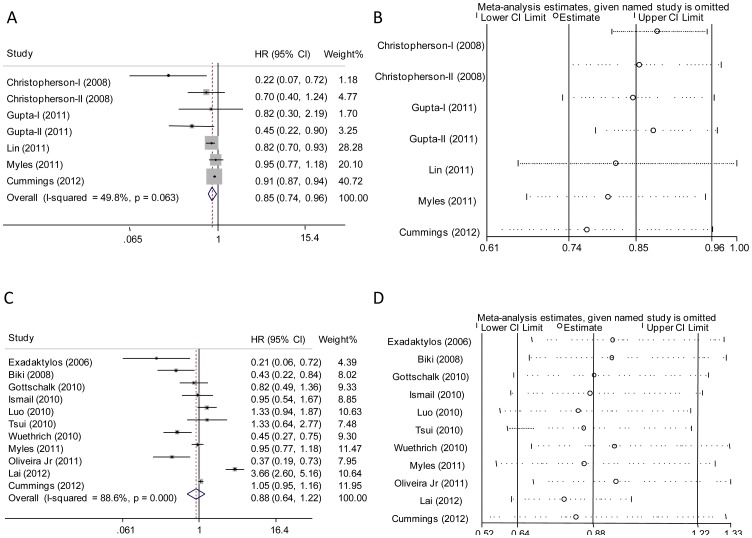
Forest plot and influence analysis of meta-analysis. In figure 2A (for overall survival analysis) and 2C (for recurrence-free survival analysis), each study is shown by the point estimate of the hazard ratio (HR) (the size of the square is proportional to the weight of each study) and 95% confidence intervals (CIs) for the HR (extending lines). Figure 2B and 2D show the influence of individual studies on the summary HR. The vertical axis indicates the overall HR and the two vertical axes indicate its 95% CIs. Every hollow round indicates the pooled HR when the left study is omitted in this meta-analysis. The two ends of every broken line represent the respective 95% CIs.

There was significant between-study heterogeneity (Table 2) in the HRs for OS (heterogeneity chi-squared = 11.9, P = 0.063, I-squared = 49.8%) with the cut-off of I-squared at 25%, so the random-effects model was used to analyze the data and demonstrated an OS benefit in favor of EA compared to GA alone (HR = 0.84, 95% CI 0.74–0.96, P = 0.013; Figure 2A). The similar result was yielded by the exclusion of the results from Cummings’s study (HR = 0.77, 95% CI 0.61–0.97, P = 0.024; Figure 2B), which had the largest sample size and may dominate the meta-analytic results. The further influence analysis also showed the robustness of our results. Because five [6], [14], [17] of the seven studies were regarding colorectal cancer, we then performed a meta-analysis specific to colon or rectal cancer (Table 2). It showed a positive association between EA and improved OS (HR = 0.65, 95% CI 0.43–0.99, P = 0.045). When meta-analysis was performed by design type, the positive association was more likely to be observed in the retrospective studies (HR = 0.86, 95% CI 0.75–0.97, P = 0.019).

**Table 2:** Pooled hazard ratios for overall survival and recurrence-free survival.

Pooled analysis	Study number	HR (95% CI)	P for difference	P for heterogeneity and I-squared
**Overall survival**				
All groups	7	0.84 (0.74 to 0.96)	0.013	0.063 and 49.8%
Excluding Cummings’s study	6	0.77 (0.61 to 0.97)	0.024	0.087 and 48.0%
In prospective studies	3	0.67 (0.37 to 1.20)	0.181	0.041 and 68.6%
In retrospective studies	4	0.86 (0.75 to 0.97)	0.019	0.137 and 45.7%
In colorectal Cancer	5	0.65 (0.43 to 0.99)	0.045	0.038 and 60.6%
**Recurrence-free survival**				
All groups	11	0.88 (0.64 to 1.22)	0.457	<0.001 and 88.6%
Excluding Cummings’s study	10	0.83 (0.52 to 1.31)	0.424	<0.001 and 89.8%
In prospective studies	2	0.98 (0.79 to 1.20)	0.817	0.388 and 0.0%
In retrospective studies	9	0.81 (0.53 to 1.26)	0.353	<0.001 and 90.8%
In colorectal cancer	3	1.06 (0.97 to 1.16)	0.217	0.266 and 24.5%
In prostate cancer	3	0.62 (0.32 to 1.20)	0.153	0.036 and 69.9%

### Association between EA and RFS

There was significant between-study heterogeneity in the HRs for RFS (heterogeneity chi-squared = 88.0, P<0.001, I-squared = 88.6%), so the random-effects model was used to analyze the data and no association between RFS and EA was observed (HR = 0.88, 95% CI 0.64–1.22, P = 0.457; Figure 2C). The similar results were yielded by exclusion of the results from Cummings’s study with the largest sample size, and the influence analysis also showed the comparable results (Figure 2D). Subgroup analyses were performed in colorectal cancer (study number = 3 [8], [17], [19]) and prostate cancer (study number = 3 [2], [7], [20]). Again, no significant association between EA and improved DFS was observed.

### Publication Bias

**Figure 3:**
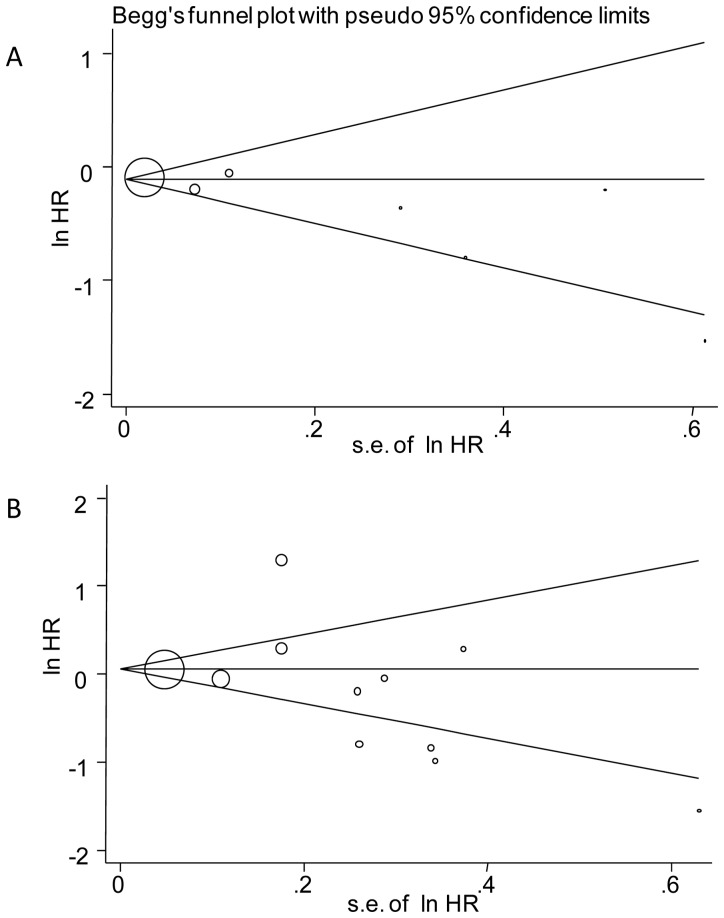
Publication bias plots. Figure 3A and 3B show the Begg’s funnel plots of studies included in the meta-analysis for overall survival and recurrence-free survival, respectively. The vertical axis represents log [HR] and the horizontal axis means the standard error of log [HR]. Horizontal line and sloping lines in funnel plot represent summary HR and expected 95% CIs for a given standard error, respectively. Area of each circle represents contribution of the study to the pooled OR.

Either graphical inspection for funnel plots or quantitative evaluation from Egger’s test indicated the absence of publication bias in OS (P = 0.241, Figure 3A) and DFS (P = 0.480, Figure 3B).

## Discussion

In the present meta-analysis, our results suggest a beneficial effect of epidural use on the OS after cancer surgery, especially for colorectal cancer. Our findings from pooled meta-analysis are consistent with most, although not all, results from currently available literature. However, epidural use did not further decrease the recurrence events of cancer.

Our meta-analysis suggests that epidural anesthesia and/or analgesia might be associated with improved OS in patients with operable cancer (especially colorectal cancer) undergoing surgery. In the Surveillance, Epidemiology, and End Results (SEER)-based study with the largest sample size, the investigators indicated a significantly beneficial effect of epidural use on all-cause mortality after colorectal cancer resection. [17] These findings, however, were in contrast to those of a prospective MASTER trial which suggested no difference in OS between the subsets of patients undergoing surgery for abdominal malignancies. [16] Besides reports in cancer surgery, meta-analyses of randomized clinical trials and population-based cohort studies of other surgeries have also demonstrated that neuraxial anesthesia might reduce mortality as well as other serious complications when compared with general anesthesia.[23]–[26] The significant effect of epidural use on reduced mortality may be consistent with the theory that there is suppression of immune defence mechanisms during surgery and in the postoperative period. [27] Other potential explanation includes stress response, hemodynamical factors, pain, and respiratory recovery. Immune compromise could affect the postoperative infection rate, healing, treatment response, as well as rate and extent of tumor dissemination during surgery. Furthermore, anesthesia and surgical procedures have been reported to suppress natural killer (NK) and T cells activity and other immune functions for a couple of days. [28]–[30] Moreover, EA is superior to GA in shifting the Th1/Th2 balance towards Th1, potentially benefiting hepatocellular carcinoma patients by promoting anti-tumor Th polarization. [31] Preservation of immune function has become a strategy to improve outcome and recent studies suggest that choosing therapeutic agents or methods that produce a sustained increase or do not decrease in the immunological function would result in a better clinical outcome.

As mentioned above, because immune surveillance is a primary determinant of cancer progression, it is logical to hypothesize that interventions aimed at reducing exposure to immunosuppressive factors would decrease patient recurrence after potentially curative cancer resection. In our study, however, this has been difficult to demonstrate. Our analysis found no association between epidural use and cancer recurrence, even in subgroup analysis for colon or prostate cancer. It seems that the reduction in mortality probably not strongly linked to cancer control. Whether negative finding represents a true association or an underpowered relationship (because of limited study number) remains to be seen. Taken together, our results suggest that the impact of epidural analgesia on cancer recurrence is minor at least. In other words, although the epidural use during surgery might influence the ultimate mortality, there should be many factors integrally influencing the final outcome and a decrease in cancer recurrence seems not to be the main mechanism by which the EA works.

Some limitations should be declared. First, our meta-analysis is limited by the nonrandomized and retrospective nature of the included studies. Second, there should be other prognostic factors not controlled in the meta-analysis. Differences in different surgical techniques, varying patient populations, changes in defining recurrence, and difficulty with long-term follow-up all hamper firm conclusions. Third, different cancer types (between and within cancer types) have differing tumor biology, whether our conclusion is suitable for all cancer types is unknown. There is only one study for some cancers, such as breast cancer and hepatocellular carcinoma. It is still too early to draw a conclusion for these cancers. Moreover, we only included studies published in English language and would introduce so called “English language bias” that may reduce the precision of combined estimates of treatment effects; this problem however exists in most currently published meta-analysis and systemic review.

In conclusion, our meta-analysis suggests that epidural anesthesia and/or analgesia is associated with improved overall survival in patients with operable cancer undergoing surgery. Our results do not support an association between epidural anesthesia and cancer recurrence. Prospective studies are needed to determine whether the association between epidural use and survival is causative.
